# Vision transformer and deep learning based weighted ensemble model for automated spine fracture type identification with GAN generated CT images

**DOI:** 10.1038/s41598-025-98518-7

**Published:** 2025-04-25

**Authors:** Sindhura D.N., Radhika M. Pai, Shyamasunder N. Bhat, Manohara M. M. Pai

**Affiliations:** 1 Department of Data Science and Computer Applications, Manipal Institute of Technology, Manipal Academy of Higher Education, Manipal, India; 2 Department of Data Science and Computer Applications, Manipal Institute of Technology, Manipal Academy of Higher Education, Manipal, India; 3 Department of Orthopaedics, Kasturba Medical College, Manipal Academy of Higher Education, Manipal, India; 4 Department of Information and Communication Technology, Manipal Institute of Technology, Manipal Academy of Higher Education, Manipal, India

**Keywords:** Computed tomography (CT) images, Deep learning (DL), Deep convolutional generative adversarial network (DCGAN), Ensemble deep learning model, Generative adversarial networks (GANs), Progressive growing generative adversarial network (PGGAN), Spine fracture, Vision transformer (ViT), Diseases, Medical research, Engineering

## Abstract

The most common causes of spine fractures, or vertebral column fractures (VCF), are traumas like falls, injuries from sports, or accidents. CT scans are affordable and effective at detecting VCF types in an accurate manner. VCF type identification in cervical, thoracic, and lumbar (C3-L5) regions is limited and sensitive to inter-observer variability. To solve this problem, this work introduces an autonomous approach for identifying VCF type by developing a novel ensemble model of Vision Transformers (ViT) and best-performing deep learning (DL) models. It assists orthopaedicians in easy and early identification of VCF types. The performance of numerous fine-tuned DL architectures, including VGG16, ResNet50, and DenseNet121, was investigated, and an ensemble classification model was developed to identify the best-performing combination of DL models. A ViT model is also trained to identify VCF. Later, the best-performing DL models and ViT were fused by weighted average technique for type identification. To overcome data limitations, an extended Deep Convolutional Generative Adversarial Network (DCGAN) and Progressive Growing Generative Adversarial Network (PGGAN) were developed. The VGG16-ResNet50-ViT ensemble model outperformed all ensemble models and got an accuracy of 89.98%. Extended DCGAN and PGGAN augmentation increased the accuracy of type identification to 90.28% and 93.68%, respectively. This demonstrates efficacy of PGGANs in augmenting VCF images. The study emphasizes the distinctive contributions of the ResNet50, VGG16, and ViT models in feature extraction, generalization, and global shape-based pattern capturing in VCF type identification. CT scans collected from a tertiary care hospital are used to validate these models.

## Introduction

The spine is made up of 33 bones known as vertebrae, serves as the primary support for the human body and enables to stand up straight, bend, and twist. The spinal canal, which protects the spinal cord, is a hollow space at the core of each vertebra. The spinal cord acts as a communication way for transmitting messages from the brain to the body. A Vertebral Column Fracture (VCF) can result in bone fragments pinching and injuring the spinal cord. The majority of VCF results from sports, falls, or car crashes. Injuries from VCF can range in severity from mild muscle and ligament strains to dislocations/serious spinal cord injury. Depending on the severity of injuries, one can feel discomfort, have trouble walking, or become paralyzed (unable to use arms or legs). Although many fractures can be treated conservatively; major fractures may necessitate surgery to align the bones.

In depth, VCF are morphologically classified as Type A, B and C. Types A, B, and C fractures are also called compression injuries, distraction injuries, and translation injuries, respectively. The type A injury is sub classified into A0-A4. The A0 injury is a minor or non-structural fracture that does not require any clinical treatment. The A1 injury is called wedge compression injury, which involves breaking of the anterior or middle endplate without involving damage to posterior vertebral wall. The A2 injury also known as split injury, involves the break of both endplates without involving damage to the posterior vertebral wall. The A3 injury is also called an incomplete burst injury, which involves a fracture of a one end plate with the posterior vertebral wall. The A4 injury, also known as a complete burst injury, involves the posterior vertebral wall and both endplates being broken. Distraction injuries are sub classified as B1-B3. The posterior tension band is damaged that continues to the vertebral body in the B1 fracture, commonly referred to as a chance fracture. Damage to the posterior tension band, which extends into the intervertebral band, is a component of the B2 injury. B3 injuries are hyper tension injuries, including injury to the anterior tension band with or without injury to the interosseous of the vertebral disc. An injury of type C, known as a translation injury, involves displacement in any direction and is not subclassified.Since there are minute differences between subtypes of fracture, identification of VCF types in the cervical, thoracic, and lumbar areas is difficult and sensitive to inter-observer variability. A DL-based automated type identification system is needed to expedite and ensure the accurate identification of VCF types from CT scans by radiologists.

Convolutional Neural Networks (CNNs) and its variants are the state-of-the-art for various computer vision applications^1^, due to their ever expanding receptive fields, which may acquire hierarchies of organized visual representations as semantics. The fundamental principle of creating effective networks in computer vision is typically considered to be the ability to capture vision semantics in images^2^. Nevertheless, long-term dependencies in images like the non-local association of visual objects are ignored by CNNs. Motivated by the success of transformers in natural language processing,Dosovitskiy et al.^3^ introduced the Vision Transformer (ViT), which describes image classification as a task of predicting the sequence of the image patch and capturing long-term dependencies within the input image. On various benchmark datasets, ViT and its variants have reached outstanding results. Transformers have gained enormous popularity and spans a broad range of computer vision tasks, including segmentation^4^ , captioning^5^ , generation^6^, detection^7^ and image classification^3^. Transformers also have a significant impact on video-based applications^8^.

Transformers have recently influenced clinical image analysis for diagnosis of diseases^9–11^ and other clinical uses. The works in^12,13^, employed ViT to quickly and efficiently treat COVID-19 patients by distinguishing COVID-19 from pneumonia utilizing CT or X-ray images. Transformers have also been used successfully for image segmentation^14^, detection^15^, and synthesis^16^, remarkably producing cutting-edge results. Both traditional DL models and hybrid models have demonstrated success in medical applications^17^.

ML and DL models have become indispensable methods for performing predictive tasks across several fields^18–21^, including healthcare^22,23^. DL models are a subclass of ML that are widely utilized to diagnose COVID-19^22,24,25^. The DL method are also used to treat pneumonia^26^. Recurrent Neural Networks (RNNs) are utilized in speech recognition^27^. While the authors of^28^ applied supervised and unsupervised techniques to describe malignancies that originated in the pancreas and lungs,^29^ used DL to identify chest pathology. Cognitive computing has also made use of ML^30^. It has long been proven that DL is capable of processing images successfully. The DL model, Alexnet^2^, demonstrated the ability to manage the large number of image processing operations.

During training, overfitting issues may occur because of the limited amount of data. Network control over the training set is often higher for smaller training data sets. Increasing the amount of training data is one of the most effective ways to reduce the overfitting of the model. Researchers employ data augmentation techniques to prevent overfitting of the model by increasing the amount of training data. Geometric translations, which include shearing, zooming, flipping, and shifting, are used to translate the images in order to expand the size of the dataset. The use of GANs for augmentation in medical applications has become increasingly common in recent years. However, the use of various GANs for the purpose of enhancing VCF type identification is not being investigated.

Sometimes, using a single model can result in biased results and overfitting. The ensemble model addresses these issues by combining the output from multiple models. From the literature, it is clear that the ensemble model performs well compared to the single model in terms of classification and anomaly identification accuracy. Over the course of the past few years, the utilization of ensemble models for the classification of medical images has grown increasingly widespread. On the other hand, there is a slight distinction between VCF types: the utilization of ensemble models for the goal of improving VCF type identification in cervical, thoracic,lumbar (C3-L5) region is not being researched. Previous research, however, solely focused on identifying compression fractures^31^ and Type A fractures^32^ not the VCFs of Types A, B, or C of (C3-L5). Therefore, the research questions addressed in this research work are as follows:

- How can an optimized weighted ensemble of Vision Transformers (ViT) and deep learning (DL) models improve VCF type classification in the C3â€“L5 region?
- How does the generation of synthetic data using extended DCGAN and PGGAN models affect the performance of VCF type identification in CT scan datasets?

Therefore, primary objective of the research is to develop an automatic DL and ViT ensemble-based VCF type identification system to assist radiologists. Research concentrates on two classes of models: ViT and DL models, which, at first, have both shown significant performance in a range of image identification applications. Pre-trained knowledge from large-scale datasets can be used by DL to overcome data size constraints and accelerate model convergence, while a relatively new paradigm called ViT allows for effective image processing with self-attention mechanisms, which makes them especially useful for medical image analysis.

The ensemble model was developed to find the best combination of DL for VCF type identification model development. ViT was also assessed for VCF-type identification. Finally, the study aims to develop the most effective weighted ensemble DL+Vit model for VCF type identification. Second Objective is to evaluate the impact of synthetic data generation through extended DCGAN and PGGAN models on improving model performance.

In short, the following is a brief list of this work’s primary contributions:

- Automatic type identification of VCF types through a weighted ensemble of best-performing DL models and ViT.
- Augmenting the limited CT VCF images through extended DCGAN and PGGAN techniques.
- Analysing the impact of extended DCGAN, PGGAN augmentation on the VCF type identification.

The remaining sections of the paper are structured as follows: The substantial relevant prior work is described in section “Literature review”, and the dataset descriptions is presented in section “Data collection”, data augmentation by extended DCGAN, PGGAN, and DL ensemble model, as well as ViT, weighted ensemble of ViT + DL for the VCF identification system, are presented in section “Methodology”. The results are shown in section “Results”. The work is discussed and concluded in sections “Discussion” and “Conclusion”.

**Table 1:** Ensemble DL models in medical applications.

Authors	Type of ensemble	Year	Contribution
P. Gifani et al.^33^	Heterogeneous Ensemble	2020	COVID-19 identification in CT images using an ensemble model.
T. Zhou et al.^34^	Heterogeneous Ensemble	2021	Deep learning ensemble models is used to detect COVID 19 in CT images.
Y. Li et al.^35^	Heterogeneous Ensemble	2021	Intelligent fault diagnosis through Ensemble Learning
A. K. Das et al.^36^	Heterogeneous Ensemble	2021	Using an ensemble model to identify COVID-19 in X-ray images.
S. Sukegaw et al.^37^	Decision Fusion	2022	Identification of osteoporosis in radiographs using an ensemble model.
A. Rath et al.^38^	Heterogeneous Ensemble	2022	Heart disease detection utilizing an ensemble model and an ECG signal.
M. Tanveer et al.^39^	Heterogeneous Ensemble	2022	Alzheimer’s disease categorization.
H. M. Rai et al.^40^	Heterogeneous Ensemble	2022	A myocardial infarction was detected.
M. Ganaie et al.^41^	Implicit ensemble	2022	Alzheimer’s disease detection via an ensemble model.
Wilson. B et al.^42^	Stacked Ensemble	2023	Classification of pancreas cancer using extreme gradient boosting
M. Roy et al.^43^	Heterogeneous Ensemble	2024	The ensemble model of ResNet variants has been fine-tuned to reduce loss while maintaining performance during chest X-ray classification.
R. F. Jader et al.^44^	Heterogeneous Ensemble	2024	The ensemble model of heterogeneous fine-tuned model to detect brain tumor in MRI.

**Table 2:** ViT in medical applications.

Authors	Type of ViT	Year	Contribution
G. S. S. Costa et al.^12^	linear attention transformers	2021	Covid19 automated detection by vision transformer in CT images.
X. Ga et al.^9^	Vision Transformer (ViT)	2021	Use sub-volumes for 3D images. With the use of a vision transformer, Covid-19 may be automatically detected in CT images.
L. Zhang et al.^10^	Swin Transformer	2021	Framework for COVID-19 classification in chest CT based on the Swin transformer.
S. Liang et al.^45^	Hybrid Model	2021	Hybrid DL framework for Covid-19 detection in 3D chest CT scans.
Y. Barhoumi et al.^46^	n-CNN-ViT Hybrid	2021	Hybrid Intracranial Hemorrhage Classification Model.
J. Li et al.^47^	Vision Transformer (ViT)	2021	An artificial intelligence platform for coronavirus diagnostics using ViT.
J. C. Than et al.^48^	Vision Transformers (ViT)	2021	The study focuses on the initial research on patch sizes in ViT for COVID-19 and the classification of diseased lungs.
Y. Xia et al.^49^	Anatomy-aware transformer with localization Unet	2021	Utilizing non-contrast CT scans for efficient pancreatic cancer screening.
S. Park et al.^50^	A novel Vision Transformers (ViT)	2021	An unique ViT for COVID-19 CXR detection that utilizes low-level CXR characteristic.
L. Tanzi et al.^51^	Vision Transformer (ViT)	2021	Classification of femur fractures.
G. van Tulder et al.^13^	Cross-view transformers	2021	Cross-view transformers are used for multi view analysis of unregistered medical images.
E. Verenich et al.^52^	Auxiliary Attention techniques for CNN	2021	Classification of Pulmonary Disease.
C. Liu et al.^53^	Vision Transformer	2021	Automatic COVID-19 Diagnosis using a Custom Transformer-Like Network.
D. Shome et al.^54^	COVID-Transformer	2021	Grad-CAM Visualization for Interpretable COVID-19 Detection in Healthcare via ViT.
K.S.Krishnan et al.^55^	Fine-tuned ViT-B/32	2021	COVID-19 detection with ViT based on chest X-rays.
S. He et al.^11^	Vision Transformer	2021	To estimate brain age, use the global-local transformer.
B.H. Kim, et al.^56^	Vision Transformer	2021	Brain graph representation by spatio-temporal attention.
H. Shin^57^	Vision Transformer	2023	Classifying Alzheimer’s disease using vision transformers.
K. Ren et al.^58^	Vision Transformer named RMT-Net.	2023	Covid-19 automated detection using vision transformer.
S. Sarraf et al.^59^	Optimized Vision Transformer	2023	Alzheimer’s Disease in MRI by optimized Vision transformer(OViTAD).
C. K. Kumar Reddy et al.^60^	Vision Transformer	2024	Multiclass classification of brain tumor in MRI by fine tuned Vision transformers.

## Literature review

In recent times, promising results have been observed in various medical applications using DL architectures. CNN is a key strategy for success in DL, especially in transfer learning and fine-tuning, which has developed with advances in technology and computer sciences and is now a crucial portion of computer vision applications. It has been applied in a variety of study areas including radiology, ophthalmology (retinal fundus images)^61–64^, as well as on chest X-rays^65,66^. In particular, in orthopedic applications, significant advancements have been made in the segmentation of spine fractures using DL methods. The reviewed studies demonstrate a range of approaches leveraging CNN architectures. For instance, attention-based multi-scale feature extraction methods have been effectively utilized in CT and MRI imaging modalities, as seen in Studies^67^ and^68^, respectively. Lightweight architectures like MobileNetv2, employed in^69^, showcase minimized computational complexity while achieving effective 3D segmentation using multi-view feature fusion. The research work^70^ further combines segmentation and recognition tasks using modified U-Net and 3D Mobile Residual U-Net (MRU-Net), providing comprehensive hierarchical feature extraction despite increased computational demands. These advancements highlight the potential of DL in orthopedic imaging, facilitating improved diagnostic precision and workflow efficiency.

Ensemble learning improves the performance of DL models by combining multiple distinct models, leading to better generalization performance and enhancing the final model’s overall performance by combining the advantages of ensemble learning with DL^71^. Numerous works in the literature demonstrate the use of DL ensemble models for medical classification and detection tasks. Table 1 lists a number of related works that use ensemble models in medical image applications.

Transformers are simple technology that can process text, speech, video, and images, making them a popular Natural Language Processing (NLP) paradigm due to their self attention mechanism. In medical imaging tasks, Vision Transformers (ViT) offer competitive alternatives to CNNs with comparable performance levels, making them a promising alternative to CNNs. The literature review Table 2 provides an in-depth assessment of numerous research initiatives using ViTs and their variants for medical imaging applications, with a focus. The table demonstrates the variety of ViT architectures and their expanding impact on improving diagnostic accuracy in healthcare. Several research have focused on COVID-19 detection, using ViTs to evaluate CT scans and chest X-rays. For example^12^ used linear attention transformers to automate COVID-19 identification in CT images, whereas^9^ used sub-volumes for 3D imaging, indicating the viability of ViTs in processing high-dimensional medical data.Similarlly, tt is evident from the literature that minimal work-in fact, no work-is using ViT for VCF type identification in the C3-L5 region.

All of these studies show that:

- There is very minimal research on GAN-powered VCF image augmentation^72,73^, which is a significant gap despite advancements in GANs in medical image synthesis.
- DL ensemble models and ViT model have promising potential in medical classification tasks but lack their usage in VCF type identification.There is a lack of weighted ensemble models of DL and ViT for type-identifying C3-L5 region VCFs. The literature also provides insightful information about how to develop ensemble models for improved and timely diagnosis of disease in the medical field, which can be used in VCF type identification. Therefore, the research gap highlights the motivation and need for the development of weighted ensemble models with GAN augmentation for VCF type identification.

## Data collection

The Computed Tomography (CT) scans precisely display the body’s skeletal structure. CT scans are widely utilized for VCF detection because they provide more precise information regarding VCFs.In this research work, VCF CT scan images were collected from a Kasturba Medical College, Manipal, Manipal Academy of Higher Education, Manipal, Karnataka, India. The study protocol was approved by the Institutional Ethics Committee (IEC) of Kasturba Medical College, Manipal, Manipal Academy of Higher Education(MAHE), Manipal, Karnataka, India, with approval number“IEC:503/2020”. All anonymized CT images were collected in accordance with relevant guidelines and regulations, adhering to the ethical standards outlined in the institutional ethics committee policies.

This retrospective study was done on a group of patients with spine fractures who got their fracture diagnosis between July 2017 and June 2020. Due to the retrospective nature of the study the informed consent was waived and was approved by the Institutional Ethical Committee of Kasturba Medical College, Manipal, Manipal Academy of Higher Education, Manipal,Karnataka, India.

**Fig. 1:**
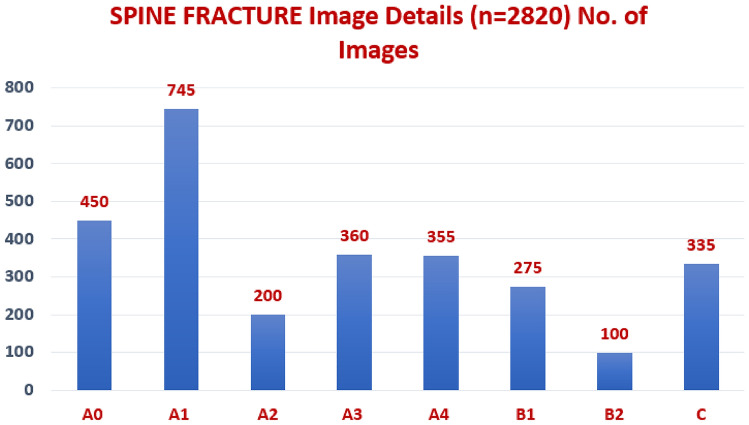
Collected VCF CT dataset description (n = 2820).

**Fig. 2:**
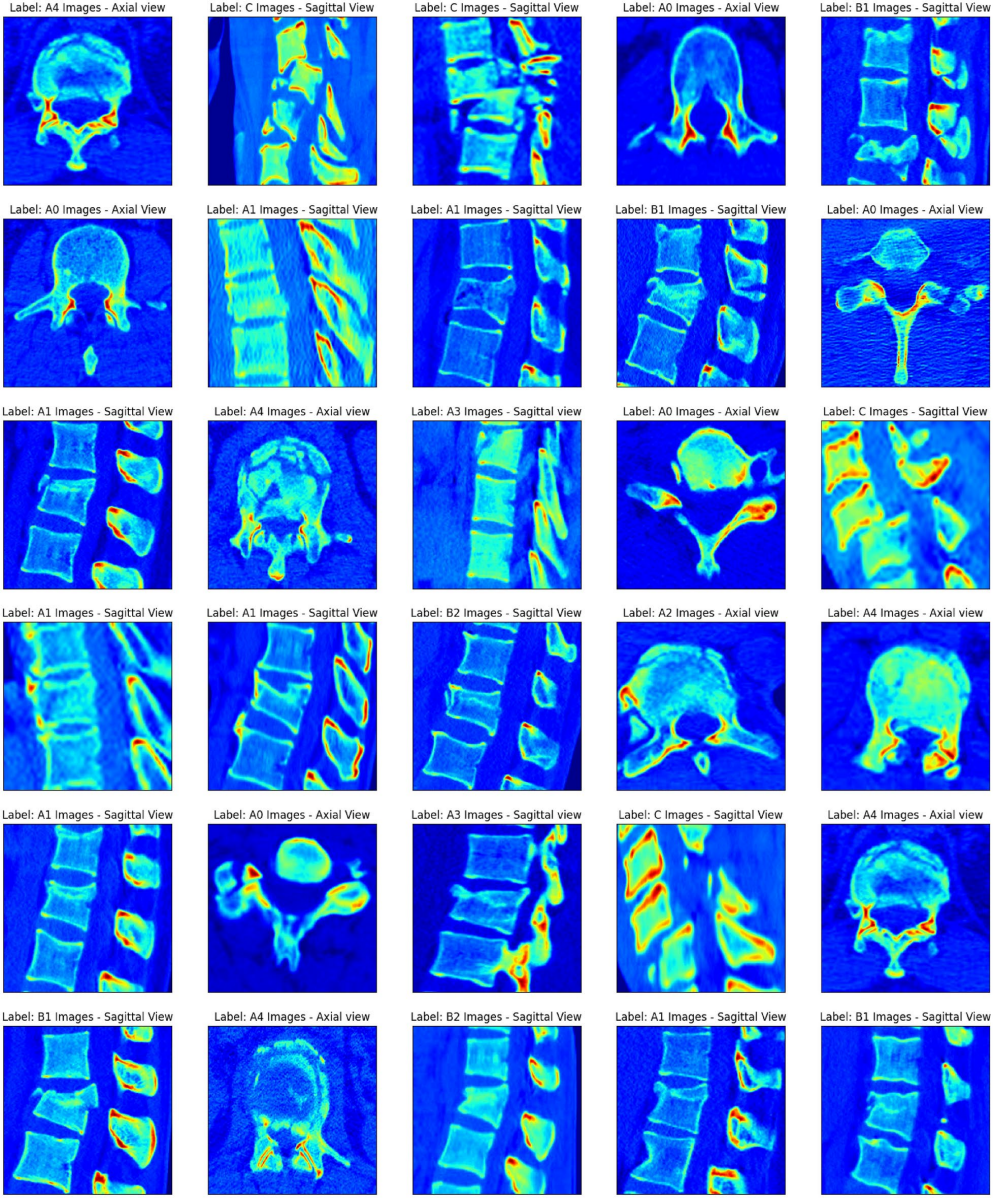
Sample VCF CT images from the collected dataset.

Retrospectively, the CT images collected prior to treatment. The following were the inclusion requirements: (1) CT scans of the patients aged between 18 and 60 with sub-axial vertebral column fractures (C3-L5); (2) CT scans of the patients who had contrast-enhanced CT scans. The following were the exclusion requirements: (1) Patients who had any type of treatment previous to the CT scan; (2) Poor-quality CT images. All of the included scans have been made anonymous.The Collected VCF CT dataset description (n=2820) may be seen in Fig. 1. The acquired instance VCF images are shown in Fig. 2.

## Methodology

**Fig. 3:**
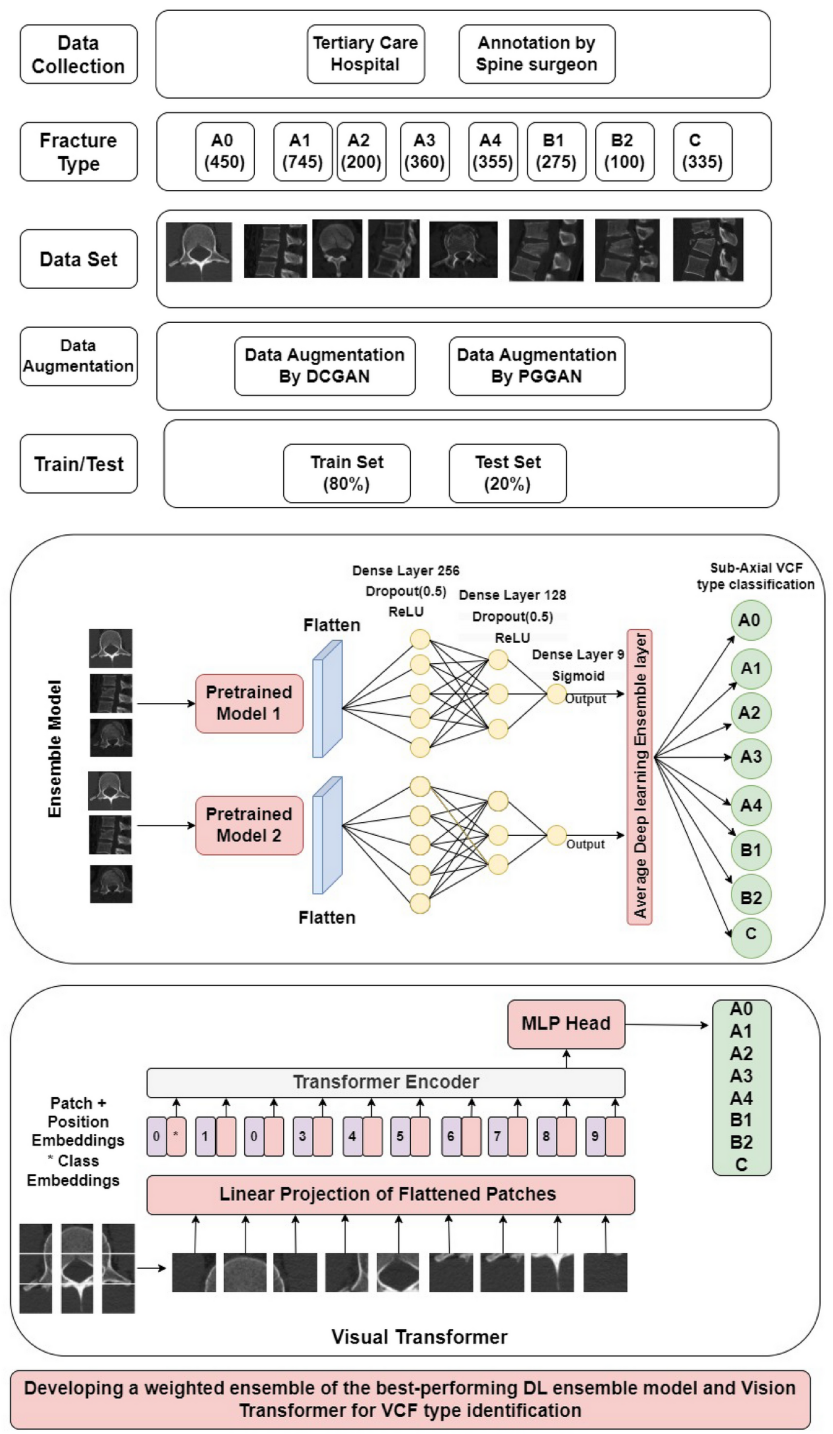
Block diagram for VCF type identification.

The sub-axial VCF CT scans were acquired from a tertiary care hospital. The acquired VCF CT scans were annotated by two spine surgeons. The interobserver reliability of annotations is present in the paper^74^. After annotation, the data is divided in the ratio 80:20 as training and testing data. The overfitting issue during the training of the models is caused by limited data. To overcome class imbalance problem, to reduce overfitting, researchers use data augmentation techniques to increase the size of the data set. Traditionally, geometric translations of images, such as shearing, zooming, flipping, and shifting, are used to expand the size of the data set. Lately, GAN-based augmentation has been used in medical applications. GAN, particularly DCGAN, and PGGAN augmentation offer a more realistic dataset than traditional techniques, allowing models to learn more representative and diverse features. This improves generalization ability by generating synthetic samples that closely resemble real data distribution. Additionally, GAN augmentation reduces the risk of overfitting by adding more unpredictability to the training process, reducing the likelihood of learning specific patterns from the small original dataset^75^. Hence, extended DCGAN and PGGAN augmentation methods are used for VCF augmentation. A block diagram for VCF type identification is shown in Fig. 3.

### Data augmentation

#### Extended DCGANs

A GAN^76^ variation called DCGAN was proposed in 2015. The DCGAN algorithm combines the unsupervised GAN method and supervised CNN learning. GANs don’t require a particular cost function to learn feature representation from input images; however, training them is unstable, and they generate noisy output images. However, in the DCGAN^77^, CNN’s structural modifications are made to expedite convergence and improve image quality. Here, extended DCGAN is developed to generate 256*256 VCF images, which contains

- Generator: The DCGAN generator comprises transposed convolutional layers. The generator (G) input is the noise vector (Z), which is taken from a uniform gaussian distribution. Numerous kinds of spine fractures may be produced by modifying the DCGAN generator and discriminator architecture. More transposed convolutional layers are added to the generator in order for the proposed expanded DCGAN to generate CT images with a resolution of 256*256. Seven transpose convolutional layers make up the generator, and they modify image dimensions as: (4*4), (8*8), (16*16), (32*32), (64*64), (128*128), and (256*256). Fractional-strided convolutions are used in place of pooling layer.Fully connected layers are eliminated from the generator’s top layers, and batch normalization is utilized to stabilize the training process.The generator network employs leaky ReLU activation throughout all levels, and the tanh function is used at the output layer.
- Discriminator: The DCGAN discriminator comprises convolutional layers. The discriminator of the proposed extended DCGAN needs to have more convolutional layers added in order to generate CT images with a resolution of 256*256. The discriminator is modified to have seven convolutional layers; strided convolutions are employed in replacement of pooling layers. There is a leaky ReLU activation. The possibility that the image is real is indicated by the discriminator’s result, which ranges from 0 to 1. Hyperparameters were tuned iteratively based on visual inspection of the generated images. Here for training, a batch size of 10 was used, along with 2000 epochs and a lr of 0.0002 .

#### PGGAN augmentation

In PGGAN, the discriminator and generator are trained incrementally^78^. Utilizing a latent space vector of size 512, it first generates realistic, low-resolution VCF pictures (4x4 pixels), after which it trains a discriminator to distinguish between actual and produced images. The generator and discriminator are then progressively enhanced by adding convolutional layers to generate VCF images in the sizes of 8, 16, 32, 64, 128, 256, and 512 [63]. Images with 512 dimensions undergo processing in batches of eight, and VCF images with 256 or less dimensions are processed in batches of sixteen. An Adam optimizer was used to train the PGGAN model. The loss is assessed using the Wasserstein loss. An NVIDIA GeForce GTX 1650 GPU was used to implement the PGGAN architecture to generate the images. Hyper parameters were tuned iteratively based on visual inspection of the generated images. The realism of the generated images was measured through visual Turing Test by the spine surgeons.^73^.

### The optimal DL models selection for VCF type identification through ensemble model development

The ensemble of DL models is a type of technique that enables the integration of many DL models to give an output that is better and enhanced than applying the single model. The generated ensemble predictive model’s purpose is to decrease variance, increase bias, or improve predictions. Normally bagging method is for reducing the variance, boosting to increase bias, stacking to improve the predictions. Sequential, parallel, homogenous, and heterogeneous ensemble are all parts of ensemble deep learning techniques.

In our work, for the purpose of identifying different types of VCFs,we limited the investigation to a number of models that were giving good results for medical image classification, such as InceptionV3, Xception, ResNet50, DenseNet121, and VGG16. The VGG16, ResNet50, and DenseNet121 were among those that produced promising results. These three models were therefore taken into account while building an ensemble model. A heterogeneous, parallel boosting ensemble method was used since the results from the chosen three models were corelating with one another.The averaging ensemble is the most commonly utilized method in the literature for integrating the predictions from the base learners. The average ensemble model enhances generalization performance by lowering the variance among the models since DL models have high variance and low bias. The ensemble model’s final output is obtained by averaging the base learners’ estimated probabilities for the classes.

Different ensemble models developed use different combinations of VGG16, DenseNet121, and ResNet50 models as base learners, each of which uses identical image data for training. Here, multiple ensemble models were developed to identify the best-performing combination of DL models for VCF type identification. Later, the best-performing model will be used with ViT for novel weighted ensemble model development.

- **Ensemble Model 1**: ResNet50+DenseNet121
- **Ensemble Model 2**: ResNet50+VGG16
- **Ensemble Model 3**: VGG16+DenseNet121
- **Ensemble Model 4**: ResNet50+DenseNet121+VGG16.

When compared to the other combination of models, the VGG16 and ResNet50 models produced results that were satisfactory for the collected VCF dataset. It is for this reason that the VGG16 and ResNet50 ensemble model for type identification is considered for weighted ensemble.

In each developed ensemble model, upon obtaining an input image for classification, each network classifies it separately. The convolution base of base learners extracted the features from the VCF CT scans and is flattened and is given to the classifier. The classifier comprises three fully connected dense layers, where the first layer contains 256 neurons, followed by a dropout of 0.6 and ReLu activation. The second dense layer contains 128 neurons, followed by a dropout of 0.6 and ReLu activation. Third layer with 8 neurons, followed by sigmoid activation.

The results of all of these models’ predictions are averaged together to produce the final predictions. Here, the‘Softmax’ function is used. The average of the predicted probability classes is calculated as shown in equation Equation 1^79^.

1 $$\begin{aligned} \ p^j_i=softmax ^j (O_i ) = frac{ (O_i^j)/(sum_(k=1)^K(exp(O_i^j)} \end{aligned}$$

$\ p^j_i$is Outcome probability of ith unit in jth base learner.

$\ O_j^i$ is Outcome of ith unit in jth base learner.

$\ K$ is Number of Classes.

**Fig. 4:**
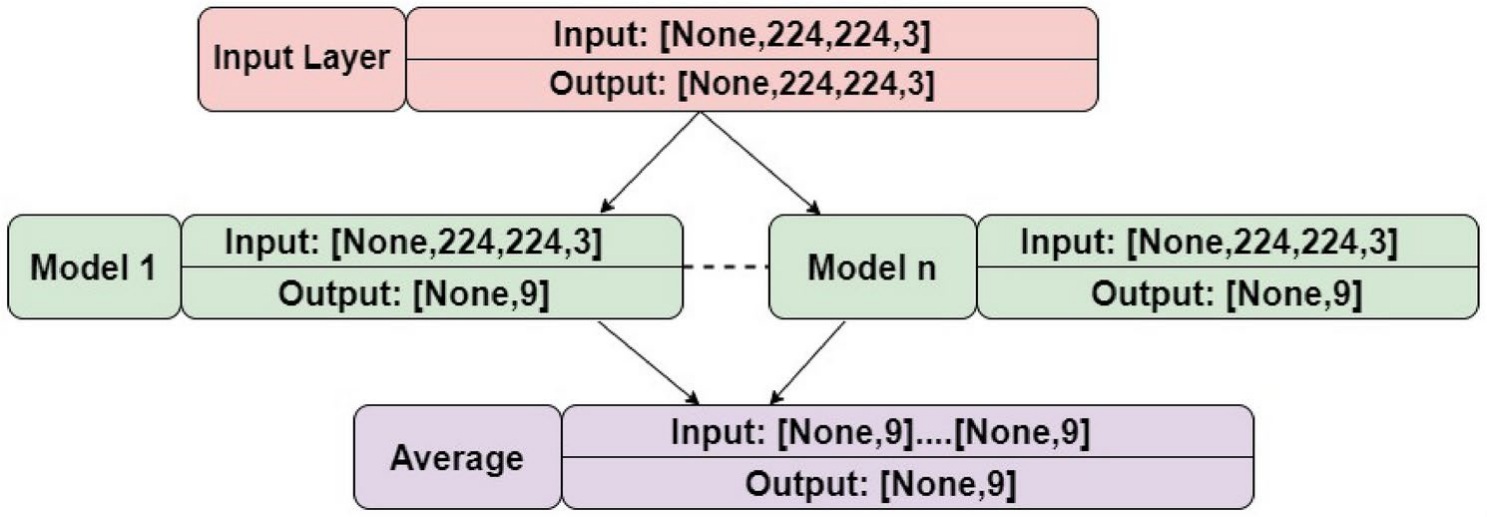
The DL based ensemble model architecture for VCF type identification.

Before finding the optimal solution for the VCF type identification problem, a variety of model configurations was tested for implemented ensemble model. Adam optimizer, categorical cross entropy loss was used in all the ensemble models. For both the training and test sets, the batch size was set to 32 was used.

Ensuring the model will function effectively on training and test data is another of the most difficult tasks in developing of DL models. The two fundamental approaches to addressing this difficulty are the collection of extensive data sets and the regularization process. Here, L2 weight regularization is used (with L2 = 0.0002). In the ensemble model, the dropout of the fully connected layer had a probability of 0.5 of stopping the activations. The hidden layer inputs of the network are normalized using batch normalization, which concentrates on accelerating optimization by minimizing internal covariate shift, which continuously modifies the distribution of the activations throughout model training. Categorical cross entropy is used as the loss function, and the learning rate is set to 0.0001. The training process took about 12 hours, and after 100 iterations, the gradient descent reached the best fit. The DL based ensemble model architecture is as shown Fig. 4

### ViT for vertebral column fracture classification

The Vision Transformer(Vit)^3^ is a kind of neural network used in computer vision application for processing images. Self-attention mechanism used in Natural Language Processing (NLP) is the base of ViT. Image processing limitations of traditional language transformers models , Recurrent Neural Networks (RNNs), CNNs, machine learning models are overcome in the ViT. In comparison to CNNs, the ViT trains data with less computational overhead and offers an accurate representation of image features.

The ViT^3^ comprises of 3 elements :

- An Eembedding layer: It is used to turn an image into a vector that transformer encoder can understand. Followed by incorporation of Location data.
- Transformer encoder: It is used for feature extraction.
- Multi-layer perceptron head: It is used to reduce the dimension of features and classify images.

It is challenging to simultaneously process a full image due to memory constraints on computers. As a result, the image is split into various patches, which are called patch embeddings. Position embeddings are inserted into patch embeddings in order to maintain positional information. Accordingly, conventional 1D learnable position embeddings are used because utilizing 2D aware position embeddings does not produce noticeable performance improvements. Lately, the class token has been added to the list of embedded patches.

The resulting sequence of embedding vectors is fed into the encoder. In more detail, the encoder consists of N identical layers stacked on top of one another, with two sublayers in each layer. The first encoder sublayer carry out the Multihead Self-Attention (MSA); the second encoder sublayer normalizes the first sublayer output and gives it to the feedforward network, which is MLP. Prior to each block, Layer Norm (LN) is applied, and each block is followed by residual connections.

ViT has a significantly lower image-specific inductive bias when compared to CNNs. Each neuron in CNNs is only linked to the neurons in its immediate vicinity.Additionally, because neurons on the same layer have identical bias values and weights, any one of them will activate if a feature of interest enters its receptive field. As a result, the feature map is comparable to feature translation, meaning that feature map can be translated in the same way as the input image.Only the MLP layers in the ViT have locality and translation equivariance characteristics. On the other hand, the self-attention layers are referred to be global since the computations carried out at these layers are not limited to a small two-dimensional region. The 2D neighborhood structure is used relatively sparingly throughout the model’s development, initially for patching the image and then during fine-tuning for adjusting the position embeddings for images of various resolutions. Beyond this, every spatial interactions between patches need to be learned entirely from scratch because the initialization time position embeddings do not contain any data regarding the 2D positions of the patches.

Large datasets are frequently used for pre-training ViT, with fine-tuning for (smaller) downstream tasks. This is done by removing the pre-trained prediction head and replacing it with a feed forward layer that occur after the input layer. It is frequently beneficial to fine-tune at a resolution greater than pre-training. The patch size remained constant when fed better-quality images, resulting in a longer effective sequence length. While the pre-trained position embeddings may no longer be relevant, the Vision Transformer can handle any sequence length (within the bounds of memory). The pre-trained position embeddings underwent 2D interpolation based on their location in the original image.

Capturing global dependencies is necessary throughout the VCF type identification process. ViT designs use self-attention methods to capture global dependencies; as a result, the ViT model is implemented to improve type identification and accurately identify VCFs. ViT architectures for VCF classification have been implemented. It has 12 layers, a MLP size of 3072, 768 hidden dimensions, and 86M parameters. This involves preprocessing the input images into patches, capturing global dependencies using self-attention mechanisms, fine-tuning the model on VCF data, and applying Extended DCGAN and PGGAN data augmentation techniques. 20% synthetic fracture images were added to the class with less CT images like A2, B1,B2,C. Then the flattening layer is added, and then the classification layers, which consist of three dense layers with 512, 256, and 8 nodes inserted with drop-out layers in between. Finally, assess the model’s performance using three metrics.

### Weighted ensemble of the best-performing DL and ViT for VCF type identification

**Fig. 5:**
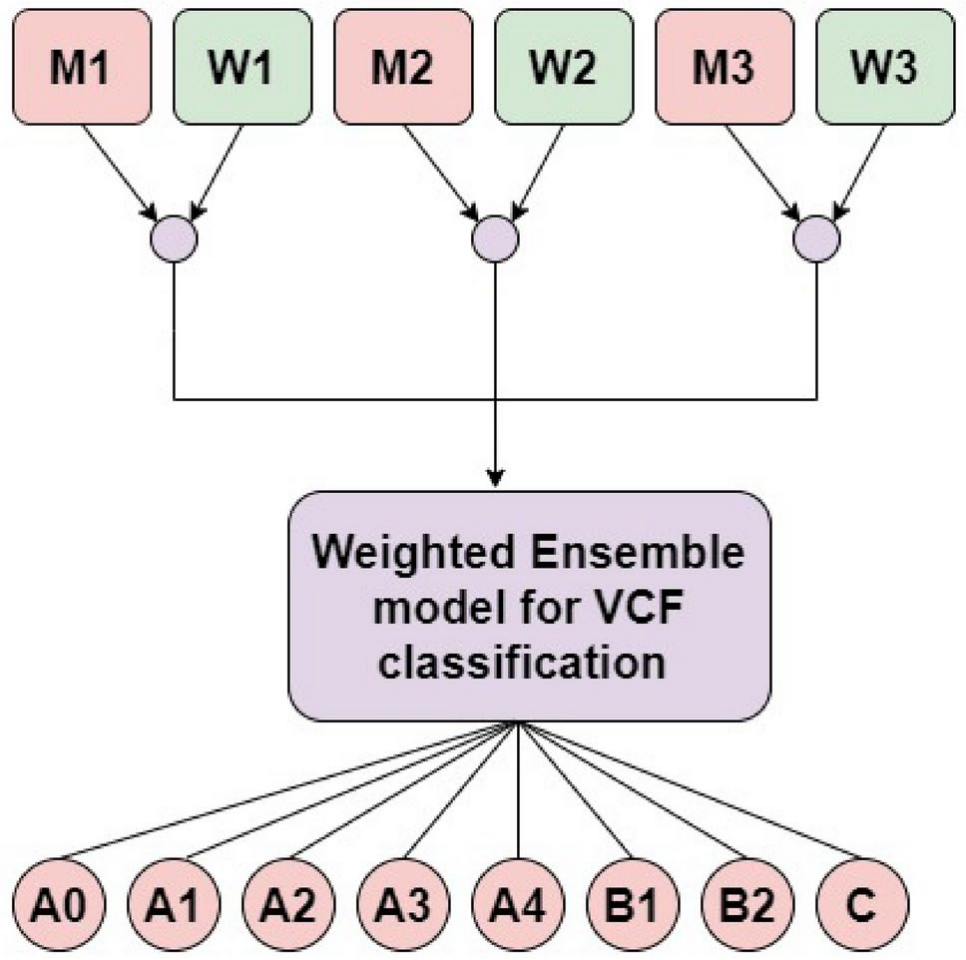
Weighted average ensemble model architecture for VCF classification.

The aim of weighted average ensemble is to improve the system’s overall accuracy through combining the trained models and allocating weights based on performance.The prediction using the weighted average ensemble approach is shown in Fig. 5.

The types of VCFs were determined using a number of models, such as InceptionV3, Xception, ResNet50, DenseNet121, and VGG16. VGG16, ResNet50, and DenseNet121 all produced noteworthy results. They were thus included in the development of ensemble models. When tested on the collected multiclass small VCF dataset, the ensemble model of VGG16 and ResNet50 performed well, as VGG16 and ResNet50 are particularly well suited at handling small datasets. Therefore, the weighted ensemble is developed using the VGG16 and ResNet50 ensemble models for type identification.

The bestperforming VGG16 and ResNet50 models are initialized using the ImageNet pretrained weights during VCF-specific pretraining, trimmed at their ideal intermediate layers, and added with a flattening layer as well as three dense layers of 512, 256, and 8 nodes, respectively, in between drop-out layers. Pilot assessments are used to determine which intermediate layers are best for the job at hand. The pretrained weights of the ViT are built using a combination of the ImageNet and Imagenet21K datasets. After that, these models are added with a flattening layer, followed by classification layers, which include three dense layers with 512, 256, and 8 nodes added in between drop-out layers. The proposed model combines the VGG16, ResNet50 DL models, and ViT using the Weighted Average approach.

The three models (VGG16, ResNet50, and ViT) were trained independently, and their results were then integrated with weights (W1, W2, and W3) whose Values ranging from 0 to 1 were assigned. The models’ priorities were taken into consideration while allocating the weights. The model (ViT) with the best performance out of the three was given the highest priority. Using a tensor dot operation and each model’s prediction value, a weight was assigned to each model. The ensemble of DL and ViT models is trained for 100 epochs using with an initial learning rate of 0.001,adam optimizer in order to minimize the categorical cross-entropy loss during VCF-specific pretraining.Model checkpoints were stored via callbacks.

## Results

The classification model performance was assessed using the following three metrics: Recall, Accuracy, and F1-score. The following definitions defined to these three-evaluation metrics

$$Recall = TP / TP + FN$$

$$Accuracy = TP + TN / (TP + TN + FP + FN)$$

$$F1 Score = 2*Precision*Recall/ (Precision + Recall)$$

**Fig. 6:**
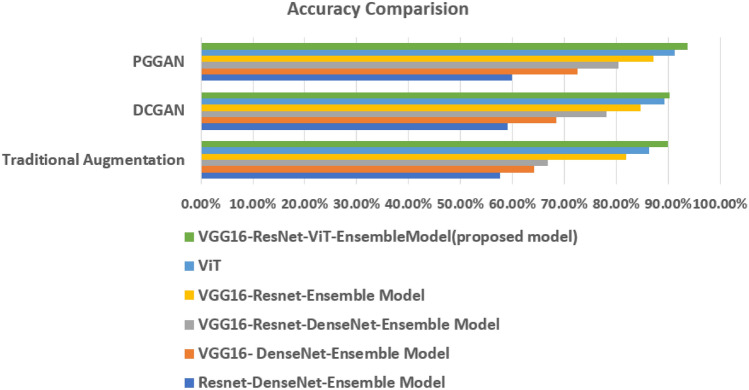
Accuracy comparison of different ensemble models in VCF type identification.

**Fig. 7:**
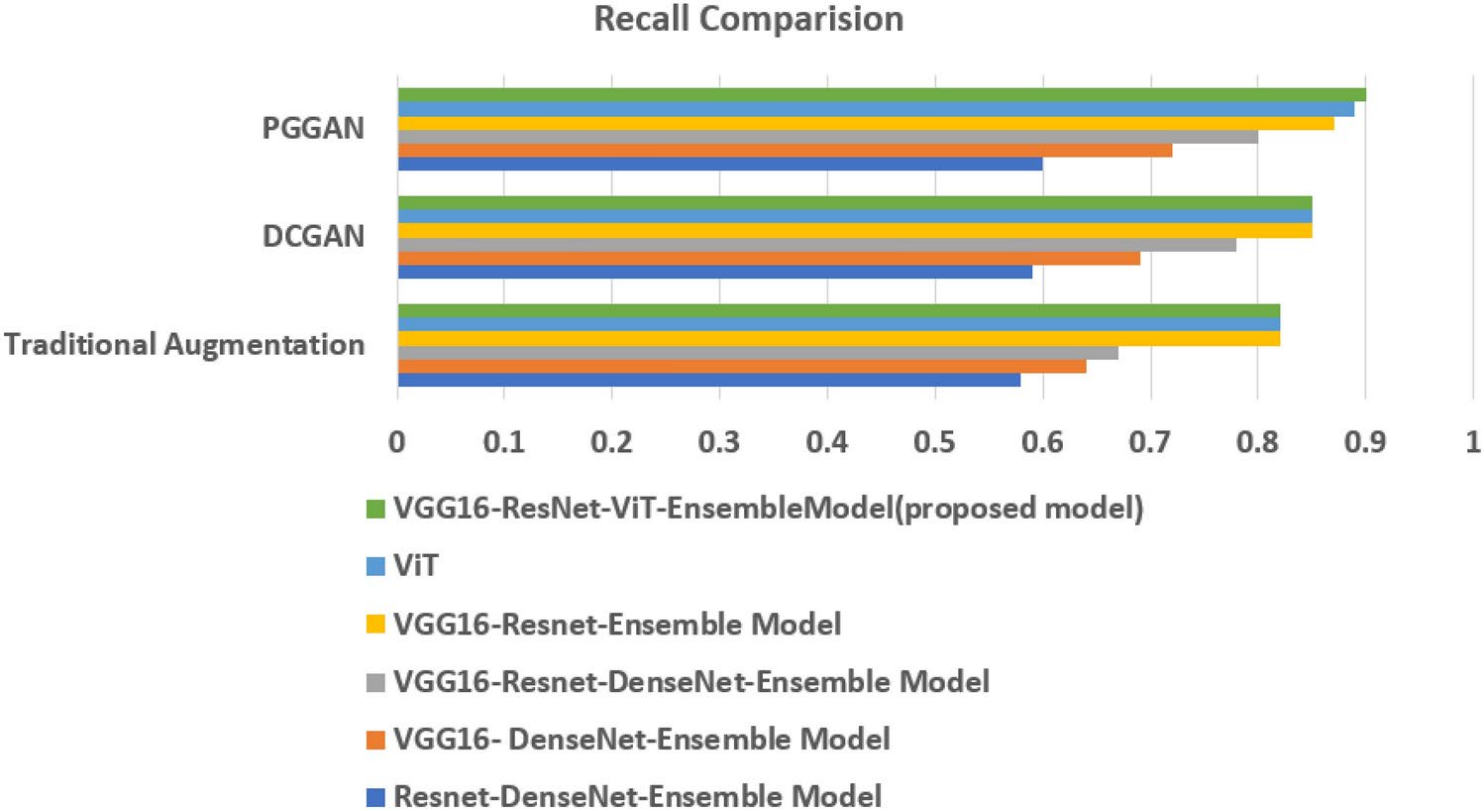
Recall comparison of different ensemble models in VCF type identification.

**Fig. 8:**
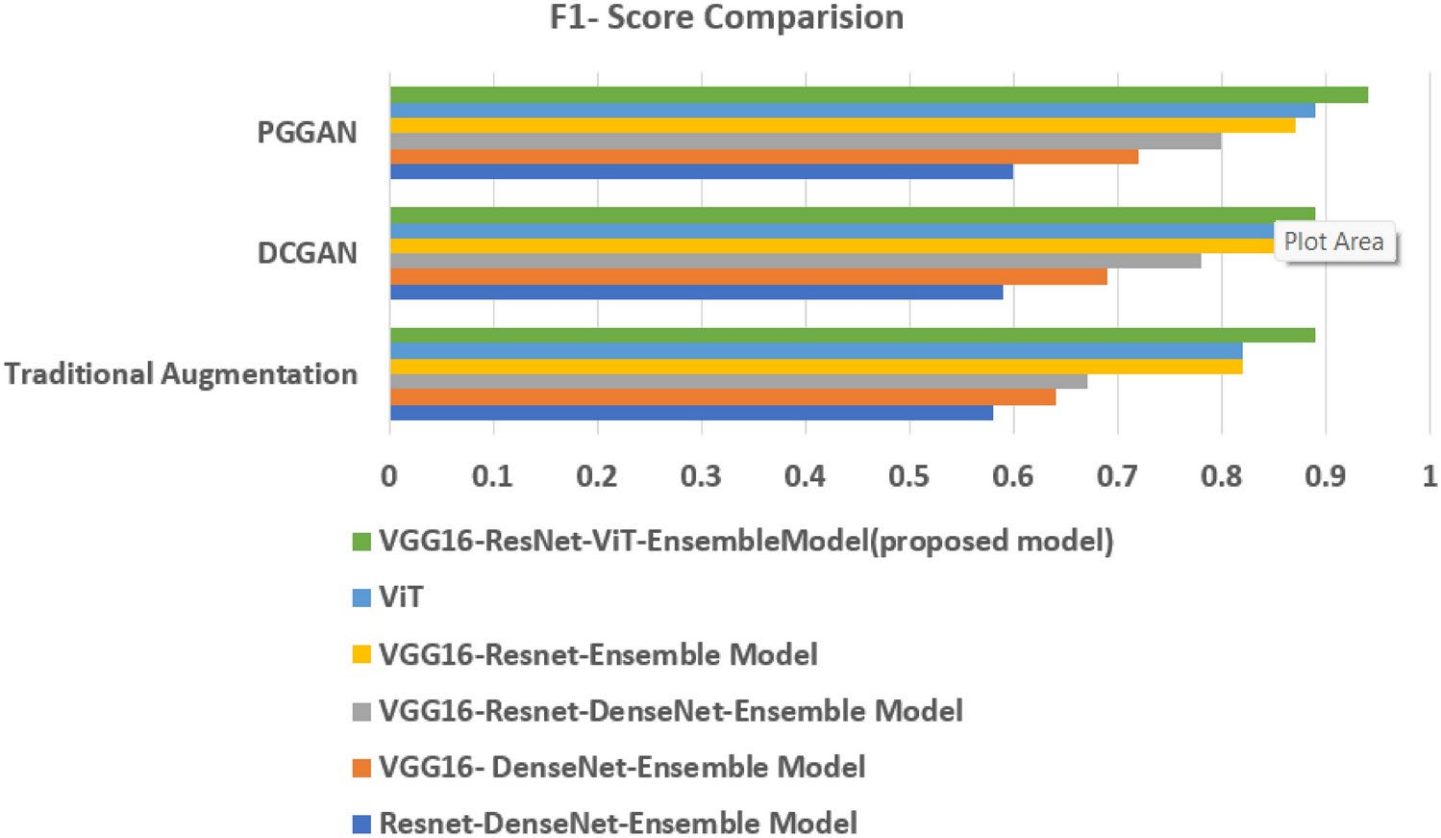
F1score comparison of different ensemble models in VCF type identification.

The Type identification Models Performance with Traditional, DCGAN, PGGAN Augmentation are presented in Tables 3,4, and 5 respectively. A comparison of performance metrics for different ensemble models is presented in Figs. 6, 7, and 8.

**Table 3:** Type identification models Performance with geometric augmentation.

Ensemble models with geometric augmentation	Accuracy (%)	Recall	F1-Score
Resnet-DenseNet-Ensemble Model	57.67	0.58	0.51
VGG16-DenseNet-Ensemble Model	64.20	0.64	0.60
VGG16-Resnet-DenseNet-Ensemble Model	66.84	0.67	0.63
VGG16-Resnet-Ensemble Model	81.83	0.82	0.81
ViT	86.28	0.82	0.82
VGG16-ResNet50-ViT-Proposed Ensemble Model	89.98	0.82	0.89

**Table 4:** Type identification models with DCGAN Augmentation.

Ensemble Models with DCGAN Augmentation	Accuracy (%)	Recall	F1-Score
Resnet-DenseNet-Ensemble Model	59.08	0.59	0.54
VGG16-DenseNet-Ensemble Model	68.49	0.69	0.63
VGG16-Resnet-DenseNet-Ensemble Model	78.13	0.78	0.72
VGG16-Resnet-Ensemble Model	84.66	0.85	0.81
ViT	89.28	0.85	0.80
VGG16-ResNet50-ViT-Proposed Ensemble Model	90.28	0.85	0.89

**Table 5:** Type identification models with PGGAN Augmentation.

Ensemble Models with PGGAN Augmentation	Accuracy (%)	Recall	F1-Score
Resnet-DenseNet-Ensemble Model	59.96	0.60	0.57
VGG16-DenseNet-Ensemble Model	72.49	0.72	0.64
VGG16-Resnet-DenseNet-Ensemble Model	80.42	0.80	0.75
VGG16-Resnet-Ensemble Model	87.13	0.87	0.82
ViT	91.28	0.89	0.85
VGG16-ResNet50-ViT-Proposed Ensemble Model	93.68	0.90	0.94

## Discussion

The research presented here is novel in that it makes strategic use of DL ensemble models and ViT, with a specific focus on the efficacy of ViT, to type identify VCF in C3-L5 region of spine. From literature it is evident that, no prior research has been conducted to identify Type A, Type B, Type C VCF in C3-L5 region. This automatic identification is to expedite patient care by cutting down on the amount of time needed for image interpretation. Models like the VGG16 and ResNet50 ensemble performed admirably in VCF type identification; however, VIT stood out. Additionally, a DL and ViT weighted ensemble technique to identify the type of VCF is developed. This enhances the effectiveness of VCF type identification.This strong result highlights how difficult VCF CT image processing issues may be solved by using DL and ViT ensemble models.

An imbalanced fracture dataset is a significant barrier to automatic VCF type diagnosis using DL. Synthetic medical images are produced using various GAN models. The GAN’s synthetic images contribute to improved automatic type identification via DL. Therefore, in this study, GAN-based methods for the generation of 2D image data sets from 2D CT images is designed and implemented.

For identifying the VCF type from CT scans, the VGG16-Resnet50-Ensemble model achieved an accuracy of 81.83%, a recall of 0.82, and an F1-score of 0.81, indicating the best combination of DL models for type identification. The results show that, as compared to a single fine-tuned base learner, the ensemble model offers better type recognition. The performance of the VGG16-Resnet-Ensemble model is significantly improved with the addition of the GAN-generated images. With extended DCGAN and PGGAN augmentation, the accuracy of VCF type identification increased to 84.66% and 87.13%, respectively.

ViT achieved an accuracy of 86.28%. ViT’s VCF type accuracy further increased to 89.28% and 91.28%, respectively, with expanded DCGAN and PGGAN augmentation. These findings highlight the vision transformers’ unique ability to identify minute patterns in VCF images, resulting in more precise VCF type identification. The self-attention mechanism is at the basis of their success, allowing these models to focus attentively on important regions, considerably improving type identification accuracy. vision transformers’ robustness and generalizability make them even more appealing in the VCF type identification.

The proposed (VGG16-ResNet50-ViT) weighted ensemble achieve outstanding accuracy. The proposed model outperformed all others, with an accuracy of 89.98%. Extended DCGAN and PGGAN augmentation improved VCF type identification accuracy to 90.28% and 93.68%, respectively. The proposed (VGG16-ResNet50-ViT) weighted ensemble model performed well because it combines the unique benefits of DL models and ViT in robustness, focusing on datasets, performance optimization, evaluation, and architecture.

Points contributing to the outstanding performance of (VGG16-ResNet50-ViT) with PGGAN augmentation are discussed in detail here:

**Fig. 9:**
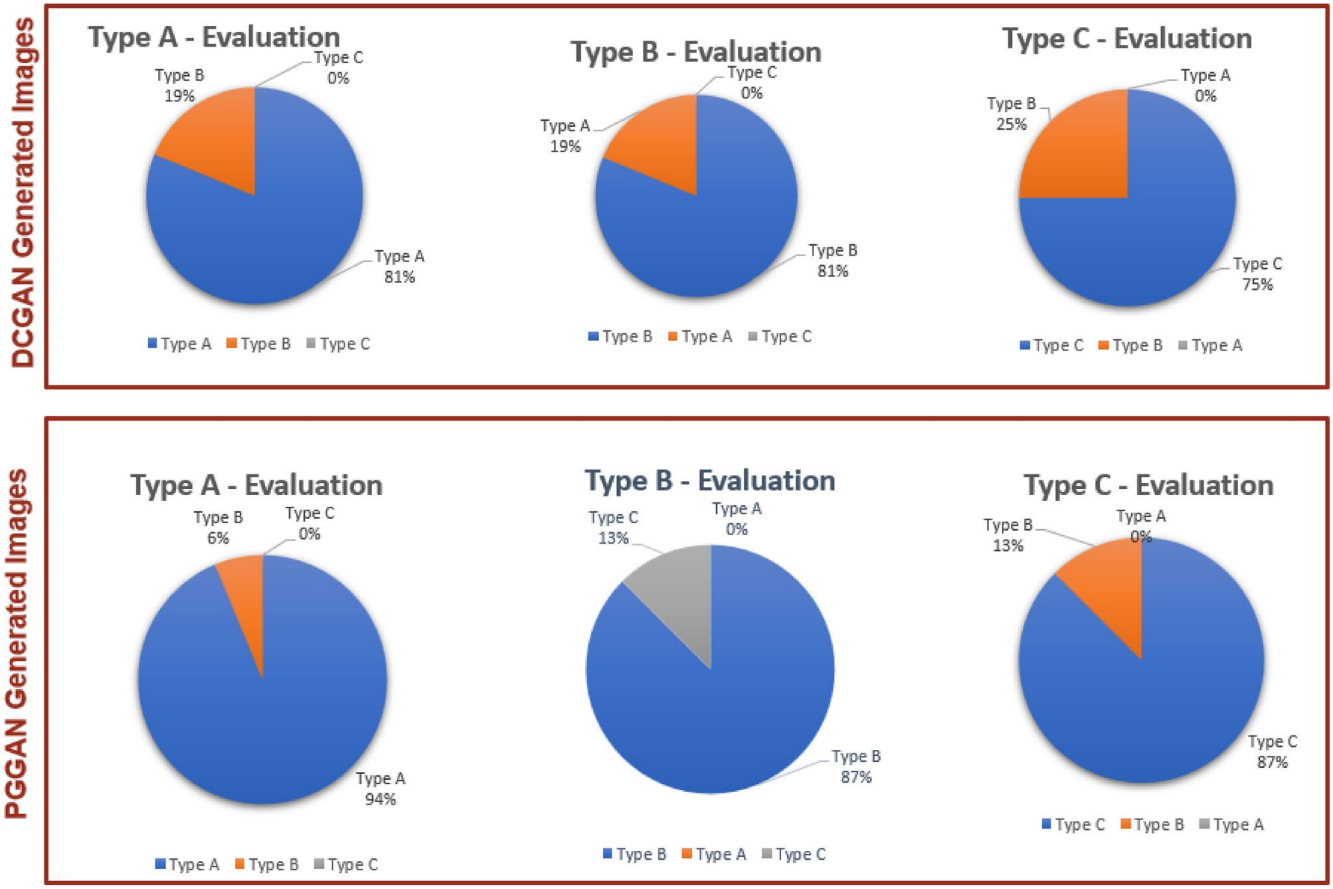
Clinical evaluation of extended DCGAN and PGGAN generated VCF images.

- DL models are widely used in image-related applications due to their extensive literature, well-established architectures, and pre-trained models. ViTs, on the other hand, can process patches concurrently, enabling faster training and inference, and are more flexible and adaptable to various datasets and applications due to their ability to handle different image sizes and aspect ratios.The developed weighted ensemble combines the advantages of both the DL models and ViT.
- The ViT in the developed ensemble model effectively collect global contextual information through self-attention techniques, representing long-range dependencies and contextual relations in VCF images. ViT can adapt to input information, adjusting receptive fields to become more resilient to changes in object orientation and size, capturing both local and global characteristics in the VCF images.
- The VGG16, ResNet50, used in the ensemble model produces feature maps by learnt features in various layers, including small-scale details in images like edges and fracture lines. ResNet-50, which is widely recognized for its residual connections, reduces the vanishing gradient issue to enable efficient feature extraction during type identification.Through selfattention processes, the ViT model excels in collecting long-range relationships in VCF images, allowing it to acquire global contextual information.
- The weighted ensemble approach in the proposed model improves performance by combining DL models and VIT with appropriate weights, reducing individual biases, and enhancing overall performance. Combining predictions with more weight for VIT helps to handle the type identification of VCF images. Weighted average ensembles enable the fusion of complementary information, providing a more holistic understanding of the dataset.
- DCGAN and PGGAN models generate synthetic data that is similar to VCF real data samples. When the real VCF dataset is supplemented with the synthetic DCGAN,PGGAN data, the diversity of the dataset increases, introducing variances that may not have been present in the initial dataset. This provides the model with a wider variety of examples to learn from. Adding synthetic data to the VCF dataset helps ease class imbalance by generating additional samples for underrepresented classes, providing more balanced training data. DCGAN and PGGANs produced synthetic data that brought diversity and randomness to the training process, improving the model’s ability to generalize to new examples. This expanded synthetic data may also help the model understand complex patterns and relationships within the different fracture types, leading to higher performance in the type identification of VCFs.
- PGGAN augmentation gives the highest accuracy in the type identification ensemble model. This is due to the fact that it learns the features in an incremental manner, beginning with a lower resolution and reaching a higher resolution. While the PGGAN is being trained, new convolutional layer blocks are being introduced in a methodical manner to both the generator and discriminator models. The gradual addition of layers enables the models to efficiently learn coarse-level knowledge and later acquire ever-finer detail. This is true on both the generator and discriminator sides of the model. while compared to DCGAN augmentation, the accuracy increased more while using PGGAN images that were generated with a high level of clarity. When 48 DCGAN and PGGAN-generated Type A, B, and C images are evaluated clinically, the same conclusion can be drawn about the situation. Figure 9 illustrates the results of the clinical evaluation taken into consideration. In comparison to the images generated by DCGAN, the images generated by PGGAN are more clear and are able to represent the fracture lines with more precision. When it comes to images created by PGGAN, the rate of type identification is consequently increasing.

## Conclusion

This study introduces a robust weighted ensemble technique for vertebral compression fracture (VCF) type identification in CT scans, leveraging the complementary strengths of VGG16, ResNet-50, and Vision Transformer (ViT) models. The ensemble architecture effectively integrates specialized texture-based features extracted by VGG16 and ResNet-50 with the advanced shape-based feature extraction capabilities of ViT’s attention mechanisms. This synergistic feature fusion enhances the model’s ability to discriminate between different VCF types, demonstrating the utility of hybrid architectures in medical imaging.

To address data scarcity and enhance model robustness, extended DCGAN and PGGAN-based data augmentation techniques were employed, generating high-quality synthetic VCF images to diversify the dataset. These augmentation methods improved the ensemble’s accuracy from 89.98% to 93.68%, illustrating the impact of synthetic data in reducing bias and improving generalization, particularly in medical imaging tasks with limited datasets.

The individual models within the ensemble contribute unique strengths that elevate overall performance: ResNet-50: Its residual connections alleviate the vanishing gradient problem, enabling efficient training and effective feature extraction for complex patterns. VGG16: The simplicity of its architecture minimizes overfitting risks, enhancing generalization across varying data distributions. ViT: Its self-attention mechanisms capture long-range dependencies and global contextual information, making it particularly suited for tasks requiring precise shape-based classification. Despite the computational complexity inherent in the ensemble approach and advanced augmentation methods, the proposed framework demonstrates significant advancements in VCF type identification. The achieved accuracy of 93.68% underscores the effectiveness of combining feature extraction methodologies with sophisticated data augmentation strategies.

Future work will explore optimizing the computational efficiency of the ensemble architecture and extending its application to other diagnostic imaging modalities, paving the way for broader adoption in clinical practice. This study highlights the potential of deep learning ensembles and generative models in addressing key challenges in medical imaging and diagnosis.

## Data Availability

The datasets analyzed during the current study are not publicly available due to privacy concerns and ethical restrictions on patient data. However, they are available from the corresponding author on reasonable request.
